# Tackling the social determinants of childhood obesity in the United States: policy brief and lessons for developed and emerging economies

**DOI:** 10.3389/fnut.2026.1786885

**Published:** 2026-07-01

**Authors:** Robert Lubajo, Olushayo Oluseun Olu

**Affiliations:** 1 World Health Organization Regional Office for Africa, Brazzaville, Republic of Congo; 2 The London School of Economics and Political Science (LSE), London, United Kingdom

**Keywords:** childhood obesity, food environment, physical activity, social determinants, United States

## Abstract

**Background:**

Childhood obesity has surged dramatically in the United States over the past three decades, with over 30% of children now classified as overweight or obese. This rising trend presents a major public health and economic challenge, with obesity-related costs rising from just over 1% of United States gross domestic product in 2005 to 3.3% in 2019 and projected to reach 4.6% by 2060 if left unaddressed.

**Analysis:**

While conventional narratives often attribute obesity to poor individual choices, this policy brief highlights the broader social determinants that shape childhood obesity across different levels of influence. Using a Socio-ecological Model Framework, the brief explores individual, interpersonal, community, and policy-level contributors, including genetic factors, psychosocial stress, family and cultural influences, school and neighborhood food environments, targeted marketing by food corporations, and regulatory gaps.

**Policy Implications:**

The brief concludes by recommending four upstream policy interventions. These include banning the sale of unhealthy foods within school premises and promoting healthy school food environments through regulation and education, implementing fiscal measures by taxing unhealthy foods and subsidizing healthier alternatives, integrating physical activity into children’s daily routines through school based and urban planning policies and Strengthening evidence systems and context-specific research. These strategies provide transferrable policy lessons for addressing childhood obesity in other settings including in low-and middle-income countries experiencing similar nutrition transitions. However, their implementation should be adapted to local health system capacities, food environments and policy contexts.

## Background

Childhood obesity is one of the most pressing public health challenges in the United States (US), with prevalence rates having tripled over the past three decades (1). Today, over 30% of children are overweight or obese, placing them at heightened risk for chronic illnesses such as type 2 diabetes, hypertension, cardiovascular disease, and mental health disorders (2, 3). The long-term implications are grave, as obese children are more likely to become obese adults, perpetuating cycles of poor health, stigma, and economic disadvantage (3). The financial burden is equally significant and continues to increase dramatically. In 2005, obesity related costs exceeded 1% of the US Gross Domestic Product (GDP) (4). By 2019, this figure had surged to 3.3% of the GDP ($705.5 billion) (5). If left unchecked, projections indicate that the cost could escalate further to 4.6% of the GDP ($2.62 trillion) by 2060 (5). The search for solutions to address childhood obesity in the US has therefore never been more urgent (6).

Historically, the dominant narrative has emphasized individual behaviors like diet and physical inactivity as the root causes of obesity. However, growing evidence highlights that these choices are deeply shaped by broader structural and environmental factors (7, 8). Agricultural and trade policies introduced in the 1970s led to the widespread availability of ultra-processed, calorie-dense foods, particularly in low-income areas (9). Aggressive food marketing, especially targeting children, has further fuelled unhealthy consumption patterns (10).

Moreover, obesity disproportionately affects children from racial and ethnic minority groups and low-income households, communities often burdened by limited access to healthy foods, inadequate opportunities for physical activity, and high exposure to unhealthy food advertising (11–13). These disparities reveal the profound influence of social determinants and systemic inequities in shaping health outcomes. Similar trends are observed in several high-income countries and low-and middle-income countries (LMICs), where childhood obesity rates have also reached alarming levels (14–16). This suggests that many of the structural factors associated with obesity in the US are not unique to that setting. In Africa, for example, this trend is also emerging due to rapidly changing lifestyles including increased urbanization, shifting dietary patterns toward energy dense processed foods and declining physical activity levels (17–20). Nevertheless, Africa and other developing contexts are characterized by the co-existence of under and overnutrition.

**FIGURE 1:**
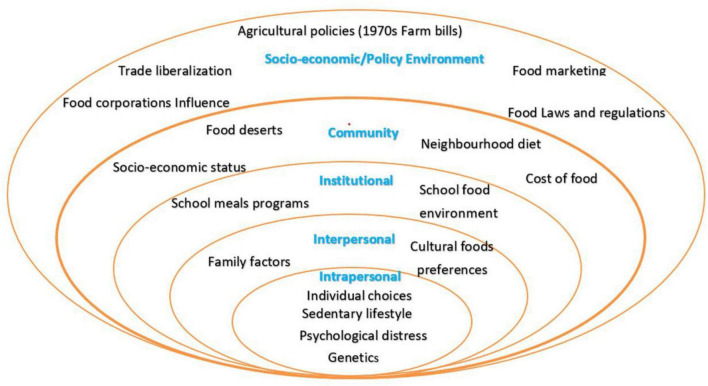
Socio-ecological model of childhood obesity in the United States.

In this policy brief, we employed a Socio-ecological Model Framework (Figure 1) to structure the analysis of childhood obesity across intrapersonal, interpersonal, community, and policy levels shifting the focus from individual behaviors to upstream determinants. The policy brief proposes priority actions aimed at reshaping both food and physical activity environments, reduce health disparities and foster healthier futures for all children.

## Sources of evidence

We gathered evidence through a targeted review of peer reviewed literature, policy reports and websites of the United States Centers for Disease Control and Prevention (CDC), United States Department of Agriculture (USDA) and the World Health Organization (WHO). We conducted a structured targeted literature search using PubMed, Google Scholar and relevant policy reports and institutional publications from national and international organizations. We guided the search using key concepts related to childhood obesity, social determinants of health, food environments, physical activity and obesity prevention policies in the US. We selected sources based on their relevance to the objectives of the policy brief and prioritized policy examples according to their national relevance, documented implementation and potential lessons for other settings. Where evidence was mixed, we placed greater emphasis on findings from systematic reviews, policy evaluations and widely cited studies while acknowledging variations in findings across contexts. The synthesis followed a narrative analysis, integrating findings across multiple sources to highlight multi-level drivers of obesity and policy responses.

## Analysis

### Intrapersonal-level determinants

At the individual level, genes play a role in body weight mostly through a combined effect of many genes (called polygenic factors) (21). Rarely, a single faulty gene can cause a disorder, and that usually shows up early in life (21). Therefore, genetics alone cannot explain the rapid rise in childhood obesity in recent decades.

Psychosocial stress and emotional distress are also significant contributors to obesity (22). This may make children to engage in emotional eating as a coping mechanism, while stress-related sleep disturbances further exacerbate weight gain. Additionally, sedentary lifestyles characterized by prolonged screen time on televisions, video games, and smartphones reduce physical activity and increase calorie intake (23, 24). Conversely, regular physical activity, adequate sleep, and early development of healthy dietary habits have been shown to play a protective role in maintaining healthy weight trajectories in children (23, 24).

### Interpersonal-level determinants

Family dynamics are central to shaping children’s weight-related behaviors. A strong association exists between parental and child Body Mass Index (BMI), as parents’ food choices, portion sizes, and general eating patterns often shape their children’s dietary habits (25–27). Over time, portion sizes consumed by children have increased and now exceed USDA’s and Food and Agriculture Authority (FDA) recommendations, contributing to caloric imbalance (28–30).

Cultural norms also play a pivotal role, particularly among ethnic minority communities. In some cultures, a larger body size is associated with health, prosperity, and good parenting (31–33). These beliefs can result in intentional overfeeding of children and resistance to health messaging about obesity. For example, among some Latin American families, a slim child may be seen as poorly cared for, reinforcing unhealthy weight norms (32, 34). In contrast, supportive parenting practices, breastfeeding, and positive family dietary norms have been associated with healthier weight outcomes and can mitigate early-life obesity risk (25, 26, 35).

### Community and institutional-level determinants

Schools are a critical institutional setting where children spend much of their time and consume significant portions of their daily food. The nutritional quality of food provided within schools and in nearby vendors can either promote or undermine healthy eating habits (36). In many cases, schools and their surroundings offer high-calorie, nutrient-poor food options that contribute to obesity (37). For example, in US schools, easy access to vending and à la carte junk foods is linked to poor students diets and increased risk of obesity (38).

Moreover, geographic disparities in food access compound the problem; “food deserts”—areas with limited access to affordable, healthy food—and “food swamps”—areas saturated with unhealthy food options—are disproportionately located in low-income and minority communities (39–41). These environments often force families to rely on ultra-processed, calorie-dense foods, contributing to unhealthy weight gain (41). Economic constraints further exacerbate the issue, making many low-income families perceive healthy food as unaffordable and thus opt for cheaper, energy-dense alternatives (41, 42). Conversely, access to healthy school meals, availability of affordable nutritious foods, and built environments that support physical activity have been shown to promote healthier behaviors at scale (35, 36).

### Socio-economic/policy level determinants

At the macro level, policy and economic structures reinforce environments that promotes weight gain, commonly referred to as obesogenic environments. The US agricultural policy, particularly the 1970s farm bills, played a pivotal role in increasing the affordability and availability of calorie-dense, nutritionally poor foods (7, 9, 43). Subsidies for crops like corn and soy led to an overabundance of cheap sweeteners and oils, which form the backbone of processed foods that dominate children’s diets (9, 43).

The food and beverage industry has capitalized on this environment through extensive marketing strategies aimed at children (3, 10). With billions of dollars spent annually on advertising junk food to young audiences, companies can influence children’s food preferences and contribute to the development of brand loyalty from an early age. The emotive language and playful imagery used in advertisements may make it more difficult for children to critically assess the nutritional value of what they consume (3, 44).

Critically, there has been insufficient regulatory response to these practices. Unlike tobacco and alcohol, unhealthy food and beverage marketing to children remains largely unregulated in the US (10). Public health experts have repeatedly called for comprehensive regulatory frameworks to limit harmful food marketing, improve food labelling, and set nutritional standards for school meals but progress remains slow and uneven (3). Some public health scholars argue that the current policy environment does not adequately protect children from commercial determinants of health, including the marketing of unhealthy foods and beverages (3, 10). On the contrary, evidence shows that strong regulatory frameworks, fiscal policies such as taxation of unhealthy foods, and subsidies for healthier options can contribute to healthier dietary behaviors and may help reduce obesity risk at the population level, although the magnitude of these effects varies across settings and interventions (45, 46).

These structural drivers are not unique to the US and are increasingly evident in other settings including in some developing economies, undergoing nutritional transition, reinforcing the global relevance of system-level approaches to obesity prevention.

### Relative contribution of socio-ecological determinants to population obesity trends

Population-level trends in childhood obesity are shaped by multiple interacting determinants operating across the socio-ecological spectrum. However, community and policy-level factors are increasingly recognized as important influences on population obesity patterns because they shape the environments within which individual choices are made. Changes in food systems shaped by agricultural subsidies, pricing, and weak regulation have increased the availability and affordability of ultra-processed foods, while sustained marketing to children has reinforced unhealthy consumption patterns. Together, these upstream factors contribute to obesogenic environments that are associated with population-wide increases in obesity (45).

Community settings, particularly schools and neighborhood food environments, further influence exposure by shaping access to food and opportunities for physical activity.

By contrast, intrapersonal and interpersonal factors influence individual behavior but do not explain temporal trends at population level. Evidence shows that interventions targeting structural determinants have greater impact at scale (46). Accordingly, policy responses should prioritize environmental and system-level change over individual behavior modification.

## Current US policies and interventions to tackle childhood obesity

Childhood obesity policies in the US are multidisciplinary and vary widely across states (47–50). Research on childhood obesity legislation in the US indicates that the type of policy proposed strongly influences its chances of being passed (51, 52). For instance, taxation-related bills are approximately eight times less likely to pass compared to those promoting behavioral interventions (48, 51), likely because behavioral interventions are more logistically and politically feasible, whereas taxation proposals on unhealthy foods face significant political resistance (51, 53). Below is a summary of the major national policies and interventions currently addressing childhood obesity in the US.

### Behavioral interventions

First, calorie labelling, introduced under the *Affordable Care Act (2010)*, mandates posting calorie information in restaurants and other food outlets (54). While intended to guide healthier choices, evidence shows that labelling has minimal influence on population-wide dietary behaviors, with many consumers ignoring or misunderstanding the information (55, 56).

Second, the *CDC School Health Guidelines (2011)* provides a comprehensive framework to promote healthy eating and physical activity in schools. However, implementation varies across districts due to funding, staff capacity and local priorities, resulting in differences in compliance and enforcement (57).

Third, the Supplemental Nutrition Assistance Program (SNAP) and its educational counterpart, SNAP-Ed, is an initiative that supports healthy dietary behaviors among low-income households in the US through nutrition assistance and nutrition education to children and families (58). However, the proposed legislative cuts threaten program continuity and future impact (59).

Fourth, the *Baby-Friendly Hospital Initiative (BFHI)*, promotes breastfeeding through the “Ten Steps to Successful Breastfeeding,” as a protective measure against childhood obesity. However, limited coverage, underserved populations, and lack of post-discharge support reduce its long-term impact (35).

Fifth, the *Healthy, Hunger-Free Kids Act (2010) (HHFKS)*, establishes state food guidelines for foods sold in school settings, promoting healthier meals for children. However, its impact is limited outside school environments (37), and implementation may vary across schools depending on resources, monitoring capacity and local compliance mechanisms.

### Socio-economic/public policy interventions

First, taxation of Sugar Sweetened Beverages (SSBs) is an intervention implemented widely in the US (60). While evidence suggests it can reduce the consumption of unhealthy foods, it has been criticized for its narrow focus only on SSBs, which may encourage substitution toward other unhealthy foods or beverages (61). Similarly, the relatively low tax rates and the absence of a uniform nationwide policy may result in inconsistent implementation judications. Taxation may also face political resistance.

Second, the regulation of unhealthy food advertising to children is an intervention that relies on voluntary pledges by the food and beverage industry, which are often violated (10). Regulatory efforts are further complicated by the rapidly evolving digital media environment, where children are increasingly exposed to marketing through social media, online gaming and other digital platforms.

Third, the *Fresh Fruit and Vegetable Program (FFVP)* funds schools to offer fresh produce snacks outside mealtimes. While it modestly reduces calorie intake and childhood obesity, challenges in implementation, produce quality, and limited impact remain (62).

Fourth, the *Special Supplemental Nutrition Program for Women, Infants, and Children (WIC)*, provides nutrition support (nutritious foods, education, breastfeeding equipment, and referrals to healthcare etc.) for vulnerable pregnant and postpartum women, infants, and children up to age five. Despite its benefits, participation remains low due to financial and operational challenges (57).

Fifth, the *“Let’s Move”* initiative, launched by former First Lady Michelle Obama through the 2008 Food and Energy Conservation Act, subsidized healthy foods in schools, providing fruits and vegetables to over 30 million students daily (63). However, it remains inadequate and doesn’t address the root causes of childhood obesity.

Finally, the *“Make America Healthy Again” (MAHA)* initiative, led by Health Secretary Robert F. Kennedy Jr., aims to combat ultra-processed foods (UPFs), seen as a root cause of childhood obesity and chronic diseases. It bans added sugars and synthetic dyes and reforms food assistance programs to exclude ultra-processed snacks. Despite its ambitious goals, the initiative faces criticism for cuts to existing school nutrition programs, limited funding, and unclear implementation (64, 65).

## Policy implications

To effectively tackle childhood obesity in the US., policy must shift from individual-focused strategies to bold, structural reforms. We recommend four integrated, population-level interventions that address childhood obesity drivers with the aim to reshape the food environment, curb industry influence, promote health equity and strengthen evidence systems and context-specific research, laying the groundwork for lasting change across communities and generations. While grounded in the UScontext, these policy actions reflect broader structural interventions that have demonstrated effectiveness across diverse settings and can be adapted to different health system and policy environments. However, their implementation and impact are likely to vary across contexts depending on health system capacity, regulatory and fiscal constraints, the role of informal food markets, and the coexistence of undernutrition and obesity in LMICs. Consequently, these recommendations should be viewed as general principles for contextual adaptation rather than direct policy transfer. The four priority policy actions are summarized in Table 1, with detailed implementation considerations described in the sections that follow.

**TABLE 1:** Summary of core policy actions to address childhood obesity in the United States.

Policy action	Target	Core mechanism	Expected population impact	Evidence strength	Equity impact	Key implementation considerations
1. Healthy school food environments	School food systems	Regulate and remove unhealthy foods; enforce nutrition standards; subsidize and provide healthy meals; integrate nutrition education	Improves dietary quality at scale; reduces early-life obesity risk and health inequalities	Strong	High	Requires sustained enforcement, school compliance and financing
2. Tax unhealthy foods; subsidise healthy diets	Food pricing and consumption patterns	Apply excise taxes on SSBs and ultra-processed foods; reinvest revenues to subsidize nutritious foods for low-income populations	Reduces consumption of unhealthy foods; shifts population diets; generates health and economic gains	Strong	High	Political resistance; potential substitution to other unhealthy products
3. Embed physical activity into daily life	Physical inactivity and built environment	Mandate school-based activity (PE, active breaks); enable active transport; strengthen community and urban infrastructure for movement	Increases physical activity levels; improves long-term physical and mental health outcomes	Moderate	Moderate–High	Infrastructure costs and cross-sector coordination
4. Strengthen evidence systems and context-specific research	Data systems and policy intelligence	Build surveillance systems; integrate obesity indicators; support implementation research and local evidence generation	Improves policy design and targeting; enables context-specific, equitable and scalable interventions	Moderate	High	Requires long-term investment and institutional capacity

***1. Ban Unhealthy Foods in Schools and Establish Policies That Promote Healthy Food Environments***

Transforming the school food environment is essential for promoting lifelong healthy eating habits. A comprehensive ban on the sale and marketing of unhealthy, UPFs within school premises linked to federal food programs should be enforced through clear school policies and national or local legislation. This combined with subsidizing healthier options in schools has proven to be cost-effective, particularly when linked to federal food programs. Simulation models suggest that nutrition standards for school meals can prevent over 345,000 cases of childhood obesity over 10 years, with savings exceeding $4.6 billion in medical costs (46). Subsidies amplify effects, especially for low-income groups, though sustainability depends on long-term political and fiscal support (46).

To ensure children have access to better options, laws and systems must also incentivize the provision and sale of affordable, nutritious foods in and around schools, through school canteens, vendors, etc., This environmental shift should be reinforced by age-appropriate nutrition education embedded in the curriculum, empowering children to make healthier food choices beyond school. Though it does not address broader food systems, targeting schools, a universal setting, ensures equitable reach and scalable impact.

Comparable approaches have shown success internationally. In the United Kingdom, strengthened school food standards have improved dietary quality among children (66, 67), while Brazil’s National School Feeding Programme has institutionalized the provision of fresh, locally sourced foods, contributing to healthier school environments and improved nutrition outcomes (68). These experiences highlight the relevance of this approach for countries expanding school feeding programmes, including many African settings.

Implementation should be school-led but nationally reinforced, aligning with USDA’s *Smart Snacks in School* (69), CDC’s *school health guidelines* (57), and *HHFKA* nutrition standards (37). Actions include regulating vendors, restricting food marketing, subsidizing healthy alternatives, and “scaling farm-to-school” programs. By leveraging existing initiatives such as the *FFVP and National School Lunch Programme*, schools can access funding streams to provide free or subsidized fresh produce, while partnerships with the *Let’s Move* initiative can enhance community engagement, awareness campaigns, and cross-sector collaboration. Strong monitoring and enforcement mechanisms, coupled with integrated nutrition education, will ensure compliance and foster sustainable, health-promoting food environments in schools. This recommendation can be implemented in a phased manner.

Potential risks to this intervention include resistance from vendors and schools reliant on food sales revenue, and uneven enforcement across districts. These can be mitigated by phased implementation, financial incentives for compliance, and dedicated monitoring systems to ensure adherence and sustain supply of affordable healthy options.

***2. Tax Unhealthy Foods and Subsidize Healthy Alternatives***

Fiscal tools can shift consumption patterns. Introducing substantial excise taxes on SSBs and UPFs will raise prices and discourage consumption, especially when paired with public messaging. For real impact, tax rates must be high enough to influence purchasing behavior. Revenues from these taxes should be reinvested to subsidize nutritious foods such as fruits, vegetables, and whole grains particularly in low-income and underserved communities.

Implementation can build on existing SSB taxes programme by standardizing excise rates nationally on sugary drinks and UPFs, ensuring rates are high enough to shift behavior. Revenues should be earmarked for programs like SNAP, SNAP-Ed, FFVP, and Gus Schumacher Nutrition Incentive Program (GusNIP) (70) to subsidize healthy foods, especially for low-income communities. In the first year of implementation, emphasis should be placed on standardizing excise taxes nationally, revenues are earmarked for expansion of the programmes. This should be followed by strengthening the enforcement, and institutionalization of subsidies for equity.

The evidence shows that SSB taxation is highly cost-effective and cost-saving (71). A nationwide 1 cent-per-ounce tax in the US was estimated to save $23.6 billion in health care costs over 10 years and generate substantial revenue, with a return on investment (ROI) ranging from $55 to $83 billion depending on coverage and enforcement (46). Taxes reduce consumption and create fiscal space for subsidizing healthier foods, making them among the strongest policy options (46). International evidence further reinforces this approach. Mexico’s national sugar-sweetened beverage tax led to a sustained reduction in purchases, particularly among low-income households (72), while the United Kingdom’s Soft Drinks Industry Levy prompted product reformulation and reduced sugar content in beverages at population level (73). Although these policies were implemented in different economic and regulatory contexts, they demonstrate how fiscal measures can influence food environments through similar pathways. These experiences provide useful experiences for LMICs undergoing rapid nutritional transition, where consumption of sugar-sweetened beverages and ultra-processed foods is increasing.

Key risks to this intervention include strong industry opposition and concerns regarding regressive effects on low-income households. this can be mitigated by clear public communication, strong political commitment, and earmarking tax revenues to subsidize healthy foods, thereby improving equity and public acceptability.

***3. Integrate Physical Activity into Children’s Daily Routines Through Supportive School and Urban Policies***

While this policy brief focuses primarily on dietary drivers of childhood obesity, insufficient physical activity remains a critical and complementary risk factor that must be addressed. Promoting regular movement among children requires more than individual motivation, it demands deliberate, systemic policies that embed physical activity into daily routines. This includes mandating quality physical education in school curricula, ensuring daily active breaks, and creating safe, accessible school playgrounds and community recreation spaces. Urban planning and transport policies should support active commuting (e.g., walking, cycling) to and from school. Such measures not only improve physical health but also enhance mental well-being and academic performance. A multisectoral approach that integrates education, health, transport, and local governance is essential to normalize physical activity as part of a healthy childhood and reduce long-term obesity risk.

Implementation of this recommendation can leverage existing federal frameworks like the *Every Student Succeeds Act* (ESSA) (74), which allows states to prioritize physical education in school accountability plans, alongside CDC’s *Active Schools* initiative to standardize daily activity requirements (57). At the community level, policies such as *Safe Routes to School* (75) and other related programs can be scaled up to promote safe, active commuting and ensure equitable access to recreation spaces. At the initial stages, emphasis should be on mandating daily breaks and after school initiatives. This should then be followed by the expansion of “safe routes” and other relevant physical activity initiatives, nationwide Physical Education (PE) adoption, urban planning for active commuting.

Globally, integrated approaches linking schools and urban design have demonstrated impact. For example, Safe Routes to School programmes in several European countries and North America have increased active commuting among children (76–78), while countries and cities such as the Netherlands and Copenhagen have embedded active transport into urban planning, contributing to sustained population-level physical activity (77, 79). These examples highlight the growing relevance of such integrated approaches in rapidly urbanizing settings beyond high-income countries.

School and community-based physical activity interventions are generally cost-effective, though with more modest returns than SSB taxes. Studies found interventions costing between $68–$140 per child annually could prevent thousands of obesity cases over a decade, with cost per QALY (quality-adjusted life year) gained well below accepted US thresholds. ROI is stronger when programs target underserved areas, where baseline activity is low (46).

Implementation risks include limited infrastructure, competing academic priorities, and weak cross-sector coordination. These can be mitigated by embedding requirements within education policy, strengthening intersectoral governance, and prioritizing investments in safe, accessible environments, particularly in underserved communities.

**4. Strengthen Evidence Systems and Context-Specific Research to Inform Policy**

Robust, context-specific evidence is essential to design, implement, and sustain effective childhood obesity interventions. While high-income countries have generated substantial evidence on obesity drivers and policy effectiveness, significant gaps remain in low- and middle-income settings, particularly across Africa, where rapid urbanization, dietary transitions, and evolving food systems are reshaping risk profiles. Strengthening national research and data systems is therefore critical to inform locally appropriate, equity-oriented policy responses.

This includes investing in routine surveillance of childhood nutrition and physical activity, integrating obesity indicators into national health information systems, and supporting implementation research to evaluate what works, for whom, and under what conditions. Schools and primary health care platforms can serve as key entry points for data generation and monitoring. In addition, partnerships with academic institutions, regional bodies, and global initiatives should be leveraged to build research capacity and facilitate knowledge exchange.

Importantly, research should not be limited to documenting risk but should focus on identifying and scaling protective factors within communities, including traditional diets, active living patterns, and social structures that support healthy behaviors. Generating such evidence will enable countries to adapt proven interventions, such as school food policies and fiscal measures, to local contexts while avoiding ineffective or inequitable approaches.

This recommendation is particularly relevant for African countries, where strengthening data systems and locally grounded evidence will be essential to guide policy prioritization, resource allocation, and long-term evaluation of obesity prevention strategies.

## Strengths and limitations

A key strength of this policy brief is its use of a socio-ecological framework to examine childhood obesity across individual, social, environmental and policy levels. By synthesizing evidence from peer-reviewed literature, policy reports and institutional documents, it translates complex evidence into practical policy actions. These strengths should, however, be considered alongside some limitations. This policy brief is based on a narrative synthesis rather than a systematic review and may not have captured all relevant studies or policy experiences. In addition, the evidence supporting obesity prevention interventions varies in quality and context, and policy effectiveness may differ across settings. While the analysis focuses on the US, lessons from this experience should be interpreted carefully and adapted as required when applied to other countries with different health systems, food environments and regulatory contexts.

## Conclusion

Tackling childhood obesity requires moving beyond individual behavior change to address the broader social and structural drivers that shape children’s food and activity environments. The recommended policies, school food regulation, fiscal measures, and physical activity integration offer scalable, equity-oriented solutions. Equally, strengthening evidence systems is essential to guide implementation, monitor progress, and adapt interventions to local contexts. While focused on the US., these interventions hold relevance for other contexts, including Africa, where similar risk factors are emerging amid rapid lifestyle transitions.
